# Adherence in chronic hepatitis B: associations between medication possession ratio and adverse viral outcomes

**DOI:** 10.1186/s12876-020-01219-w

**Published:** 2020-05-07

**Authors:** Nicole L. Allard, Jennifer H. MacLachlan, Anouk Dev, James Dwyer, Geeta Srivatsa, Timothy Spelman, Alexander J. Thompson, Benjamin C. Cowie

**Affiliations:** 1 WHO Collaborating Centre for Viral Hepatitis, Victorian Infectious Diseases Reference Laboratory, Royal Melbourne Hospital, at the Peter Doherty Institute for Infection and Immunity, Melbourne Victoria, 3000 Australia; 2 University of Melbourne, at the Peter Doherty Institute for Infection and Immunity, Victoria, 3000 Australia; 3 Department of Gastroenterology, Monash Health, and Monash University, Victoria 3168 Clayton, Australia; 4 Mercy Hospital for Women, Victoria, 3084 Australia; 5 Western Health and Footscray Hospital, Footscray Victoria, 3011 Australia; 6 Burnet Institute, Melbourne, 3000 Australia; 7 St. Vincent’s Hospital, Melbourne, 3000 Australia

**Keywords:** Adherence, Antiviral therapy, Hepatitis B, Medication possession ratio

## Abstract

**Background:**

Antiviral therapy for chronic hepatitis B (CHB) is effective and can substantially reduce the risk of progressive liver disease and hepatocellular carcinoma but is often administered for an indefinite duration. Adherence has been shown in clinical trials to maximize the benefit of therapy and prevent the development of resistance, however the optimal threshold for predicting clinical outcomes has not been identified. The aim of this study was to analyse adherence using the medication possession ration (MPR) and its relation to virological outcomes in a large multi-centre hospital outpatient population, and guide development of an evidence-based threshold for optimal adherence.

**Methods:**

Pharmacy and pathology records of patients dispensed CHB antiviral therapy from 4 major hospitals in Melbourne between 2010 and 2013 were extracted and analysed to determine their MPR and identify instances of unfavourable viral outcomes. Viral outcomes were classified categorically, with unfavourable outcomes including HBV DNA remaining detectable after 2 years treatment or experiencing viral breakthrough. The association between MPR and unfavourable outcomes was assessed according to various thresholds using ROC analysis and time-to-event regression.

**Results:**

Six hundred forty-two individuals were included in the analysis. Median age was 46.6 years, 68% were male, 77% were born in Asia, and the median time on treatment was 27.5 months. The majority had favourable viral outcomes (91.06%), with most having undetectable HBV DNA at the end of the study period. The most common unfavourable outcome was a rise of < 1 log in HBV DNA (6.54% of the total), while 2.49% of participants experienced viral breakthrough. Adherence was linearly associated with favourable outcomes, with increasing risk of virological breakthrough as MPR fell. Decreasing the value of MPR, at which a cut-point was taken, was associated with a progressively larger reduction in the rate of unfavourable event; from a 60% reduction under a cut-point of 1.00 to a 79% reduction when the MPR cut-point was set at 0.8.

**Conclusion:**

Lower adherence as measured using the MPR was strongly associated with unfavourable therapeutic outcomes, including virological failure. Optimising adherence is therefore important for preventing viral rebound and potential complications such as antiviral resistance. The evidence of dose-response highlights the need for nuanced interventions.

## Background

Chronic hepatitis B (CHB) is responsible for increasing mortality and morbidity worldwide with liver cancer related mortality increasing globally, as the population living with hepatitis B infection ages, attributable mortality from other causes of death falls [1] . In 2015, the World Health Organisation (WHO) set ambitious targets for CHB including 80% of eligible people receiving treatment and a 65% reduction in attributable mortality by 2030 [2]. Measuring viral suppression in people living with CHB is one of the 10 core indicators in the monitoring and evaluation framework for viral hepatitis produced by the WHO, and a measure of retention in care and adherence to treatment for people receiving antiviral therapy [2, 3].

Antiviral treatment is effective at both preventing progressive liver damage and liver cancer in people living with CHB who have active viral replication and ongoing liver inflammation, or who have established cirrhosis [4, 5]. Effective treatment results in a low or undetectable viral load, accompanied by normalization of hepatic transaminase levels (such as alanine aminotransferase, ALT). This can be achieved by using recommended first line antiviral agents entecavir or tenofovir, which are well tolerated, have high barriers to development of antiviral resistance, can reduce the risk of cancer by up to 75% over several years and can reverse established cirrhosis [4–6]. The optimal duration of antiviral treatment is yet to be defined, but treatment, once initiated, is most often considered to be indefinite or life-long [5]. Commencement of antiviral treatment is only a first step; as is the case for other chronic diseases requiring ongoing treatment and adherence to therapy is required to ensure effectiveness and to prevent viral breakthrough and the potential development of resistance [7].

Treatment adherence is a dynamic process in individuals and periods of poor adherence may result from temporary or more long-term disengagement from care due to competing social pressures, moving place of residence, difficulty attending clinical appointments and/or pharmacies, or interruption of supply of medication due to financial pressures, or day to day pressures that result in irregular dosing [8]. Poor adherence is also more common when the condition being treated is asymptomatic and the treatment regimen results in no overtly identifiable sense of improvement in health status [8–10]. Adherence is improved in people who have knowledge of their condition and in older individuals, and when the perceived severity of the condition is higher [8].

With new global goals to increase numbers of people with CHB on treatment, assessing and maximizing adherence will become an increasing challenge in all settings. A recent systematic review including 30 studies with different adherence measures estimated overall adherence to treatment in CHB with oral therapy was 75% and was similar in both high and low income settings [11]. Factors that have been shown to be related to poor adherence have included forgetting and change to routine, younger age, higher physician turnover and recent initiation of therapy [11–15]. Adverse reactions to medication can also result in poor adherence however antivirals for CHB have a low side effect profile and rarely adverse outcomes [16]. In a previous study we reported low adherence in 1026 participants was associated with age less than 35 years, inconsistency in clinical care and hospital site but not with type of oral antiviral prescribed [13].

Adherence can be measured by self-report, physician report, measurement of drug levels (dependent on assay availability), direct measurement with memory cap bottles and indirectly through pharmacy data [17]. Pharmacy adherence measures (PAMS) estimate medication in hand during an observed treatment period, can be easily calculated from pharmacy records and are less likely to have bias inherent in self-assessment or physician estimates, but represent only the maximal possible adherence during a time-period; actual administration of dispensed medications is not monitored [18]. Medication possession ratio (MPR) has been most commonly used as a measure of PAMS.

$$ \mathrm{Medication}\ \mathrm{possession}\ \mathrm{ratio}\ \left(\mathrm{MPR}\right)=\frac{\mathrm{Number}\ \mathrm{of}\ \mathrm{pills}\ \mathrm{dispensed}}{\mathrm{Number}\ \mathrm{of}\ \mathrm{days}\ \mathrm{in}\ \mathrm{time}-\mathrm{period}} $$

In the treatment of HIV with highly active antiretroviral therapy adherence, the MPR has been correlated with viral outcomes and mortality [18]. In the context of CHB, an MPR cut-off to determine poor adherence of 0.90–0.95 has previously been arbitrarily chosen without clear reference to viral outcomes and/or mortality and morbidity [12, 19].

In Australia about 6% of all people living with CHB are receiving treatment with oral antivirals, predominantly entecavir (45%) and tenofovir (28%) [20, 21]. Antiviral medication has primarily been dispensed by public and private hospitals at a cost to the individual of US$6–$39 per 2 months of supply. There has been limited prescribing in the community context. An estimate of adherence or the number of people taking treatment who are virally suppressed or adherent to therapy has not yet been incorporated into the cascade of care at a national or jurisdictional level [20, 22].

The aims of this multicentre study were to evaluate virological outcomes in Australian setting, determine the proportion of patients with adequate viral suppression on treatment and to establish an evidence-based MPR definition of poor adherence based on the incidence of poor viral outcomes in patients receiving long term oral antiviral therapy for CHB.

## Methods

This study was a retrospective analysis of records of patients dispensed antiviral monotherapy for CHB from 4 tertiary referral hospitals in Melbourne, Australia between 2010 and 2013. Pharmacy and pathology data were linked using hospital record number. Participants were included if they were prescribed antiviral monotherapy with > 3 months of treatment and **≥** 2 hepatitis B virus DNA or viral load (HBV DNA) tests recorded in the hospital pathology service which was the minimum needed for classification of a viral outcome. The classification scheme for unfavourable or favourable viral outcomes is as presented in Table 1. As a sensitivity analysis, non-transient rise was classified as a favourable outcome and the impact assessed (see Appendix).

**Table 1:** Viral outcome categories for HBV treatment

Favourable viral outcomes:	
Fully suppressed	HBV DNA undetectable for study period OR HBV DNA became undetectable with no further rise
Adequate viral suppression	Where treatment duration was < 2 years, HBV DNA decreased > 1 log10
Transient rise	a single instance of increased DNA level < 150 IU/ML with return to undetectable at next test
Unfavourable viral outcomes:	
Not suppressed	Detectable HBV DNA after 2 years on treatment
Non-transient rise	< 1 log rise in HBV DNA which was not a transient rise
Viral breakthrough	≥1x log_10_ rise in HBV DNA level from nadir which was not a transient rise

MPR values were calculated from records of medication dispensed from the first pharmacy visit to the date immediately after or at the same time as the event was observed, as pharmacy pick up dates did not always align with date of pathology tests. For those who were fully suppressed throughout the time-period, the MPR was calculated for the whole observed period. Participants could have more than one event recorded with separate calculations made of cumulative MPR prior to each event.

### Statistical analyses

Categorical variables were summarised using frequency and percentage. Continuous variables were summarised using mean and standard deviation (SD) or median and inter-quartile range (IQR) as appropriate. As individual patients were permitted to contribute multiple unfavourable outcomes across the observation period, an Andersen-Gill time-to-multiple event model was used to study associations between MPR and unfavourable viral outcomes. Hazard proportionality was assessed via analysis of scaled Schoenfeld residuals. Results are presented as the individual hazard ratio of unfavourable for each MPR cut off category (≥0.80; > 0.90; > 0.95; 1.0; > 1.05). All multivariable models were assessed for interactions between explanatory variables. MPR values of < 1 indicated inadequate supply for the time period or less than one tablet per day with < 0.80 = 80% tablets per number of days and 0.90 90% etc. Values > 1.0 or greater > 1.05 indicated over supply of pills dispensed per number of days in the time period. MPR was analysed with Youden analyses as a continuous variable to ascertain a possible cut off point. Further analyses of previously used categorical values of MPR to identify performance of those values of MPR in discriminating the outcome variables and was performed with receiver operating characteristic (ROC) curve, sensitivity, specificity and positive and negative predictive values (PPV and NPV). For all analyses, *p* < 0.05 was considered significant. All analyses were conducted in R (R Foundation for Statistical Computing, Vienna, Austria).

Ethics approval was granted by Human Research Ethics Committees at Melbourne Health, Monash Health, Western Health and St. Vincent’s Hospital to access de identified pharmacy records and internal patient management software for demographic information.

## Results

### Patient characteristics

Six hundred forty-two individuals’ records were included in the final analysis representing 1234 patient-years of antiviral treatment. Three hundred eighty-four individuals were excluded for insufficient data (< 2 viral loads recorded in the hospital pathology services during the period on treatment). There were no differences in age, sex distribution or adherence pattern in the excluded records (data not shown). The median time on treatment during the study period was 27.5 months (IQR 13.5–32.9) and the median number of viral loads performed 4 (IQR 3–6). Most (91.7%) of the patients on antiviral medication were born overseas, with 77% of the total born in Asia (Table 2). In total, 550 patients (85.7%) were on first-line oral antiviral therapies (either entecavir 0.5 mg or tenofovir 300 mg).

**Table 2:** Patient characteristics

	Number(%)	Median age (IQR)
Gender		
Male	441(68.69)	46.55 (38.00–56.31)
Female	201 (31.31)	46.66 (37.00–56.86)
Region of birth		
Asia	496 (77.26)	46.00 (31.00–55.09)
Europe	49 (7.63)	61.00 (53.00–68.39)
Australia	45 (7.01)	44.00 (31.00–55.60)
Africa	34 (5.30)	40.76 (33.52–47.34)
Other	10 (1.56)	45.00 (53.00–59.39)
Not recorded	8 (1.25)	

### Viral outcomes

Favourable viral outcomes were seen in 91.06% of individuals (Table 3). The majority either were undetectable for the full treatment period (48.85%) or became undetectable (26.25%) by the end of the treatment period. Of those with unfavourable outcomes (8.94% of the total), the most common was a non-transient rise (6.54% of the total), while 17 (2.54%) experienced viral breakthrough. Forty participants had 2 events recorded during treatment period MPR was calculated for the period prior or up to each event. The proportion of individuals with favourable outcomes was similar across all drugs used (75.00–92.31%, Table 2), with no evidence of difference in the proportion for entecavir vs. tenofovir (*p* = 0.30) or for first line therapy vs. older drugs (*p* = 0.38).

**Table 3:** Favourable and unfavourable viral outcomes by drug category

				Favourable outcomes^a^ n. of events (% of total outcomes by drug)				Unfavourable outcomes n. of events (% of total outcomes by drug)			
Drugs	Individuals n (% of total)	Individuals with 2 events	Fully suppressed (undetectable for study period)	Fully suppressed (became undetectable)	Transient rise	Adequate viral suppression	Total favourable outcomes	Viral breakthrough 1 log^10^ rise	Non-transient rise	Not suppressed	Total unfavourable outcomes
All	642	40	340(48.85)	179(26.25)	55(8.06)	47(6.89)	**621(91.06)**	17(2.49)	42(6.54)	2(< 0.01%)	**61(8.94)**
First line therapy											
Entecavir 0.5 mg	407(63.40)	22	216(50.35)	118(27.21)	35(8.16)	27(3.96)	**396(92.31)**	8(1.86)	24(5.90)	1(<.01%)	**33(7.69)**
Tenofovir	143(22.27)	11	61(39.61)	48(31.17)	10(6.49)	19(2.79)	**138(89.61)**	6(3.90)	9 (6.29)	1(< 0.01%)	**16(10.39)**
Other											
Entecavir 1 mg	29(4.52)	1	16(53.33)	2(10.00)	6(20.00)	1(3.33)	**26(86.67)**	2(6.67)	2(6.90)	0(0.00)	**4(13.33)**
Older drugs											
Lamivudine	53(8.26)	4	40(70.18)	8(14.04)	4(7.02)	0(0.00)	**52(91.23)**	1(1.75)	4(7.54)	0(0.00)	**5(8.77)**
Adefovir	10(1.56)	1	7(58.33)	2(20.00)	0(0.00)	0(0.00)	**9(75.00)**	0(0.00)	3(25.00)	0(0.00)	**3(25.00)**

^a^See Table 1 for definition of favourable and unfavourable outcomes

### MPR and association with virological outcome

**Fig. 1:**
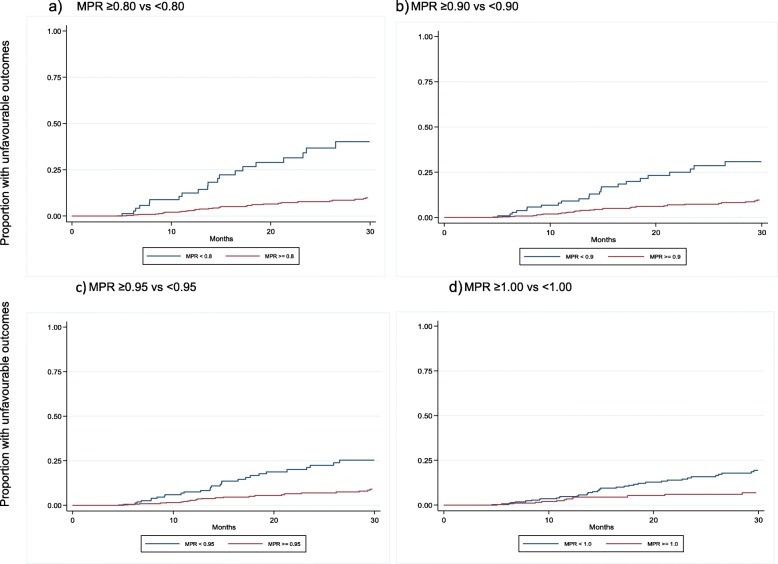
**a**-**d** Proportion of individuals experiencing unfavourable outcomes over time according to MPR cut-off points

An MPR of < 1.0 was seen in 346 individuals (53.89% of the total). In the time to event analysis, the hazard of unfavourable outcomes was increased with a lower MPR (Table 4). The proportion of individuals experiencing unfavourable outcomes over time are presented in Fig. 1a-d which demonstrate the differential outcomes according to MPR cut-off. An MPR of ≥1.0 was associated with a 60% reduction in risk of unfavourable outcomes compared to those with an MPR of < 1.00, while an MPR ≥0.80 reduced risk by 79% compared to an MPR of < 0.80. No evidence was seen of an association between an MPR ≥1.05 and viral outcomes.

**Table 4:** Hazard ratio for unfavourable events for each MPR cut off threshold, and interpretation of risk reduction of unfavourable events

MPR threshold comparison	Hazard ratio for unfavourable events with MPR above threshold (95% CI) *p*-value	Interpretation of risk reduction of unfavourable events with MPR above threshold
≥0.80 vs < 0.80	0.214 (0.12–0.37) < 0.001	79%
≥0.90 vs < 0.90	0.292 (0.17–0.50) < 0.001	71%
≥0.95 vs < 0.95	0.334 (0.20–0.57) < 0.001	67%
≥1.00 vs < 1.00	0.398 (0.22–0.73) 0.003	60%
≥1.05 vs ≤1.05	0.600 (0.29–1.44) 0.287	No evidence of association

The empirical cut-point on the Youden analysis for MPR was 0.98, with sensitivity and specificity at that threshold of 41% and area under ROC 0.41. Further analyses of sensitivity, specificity, PPV and NPV according to MPR categories are presented in Table 5.The specificity demonstrates the ability of MPR to predict unfavourable outcomes increased with higher MPR cut off points, however PPV was low across all MPR cut-point thresholds, which reflects the finding that the majority of individuals with an MPR below threshold did not experience an unfavourable viral outcome. NPV, conversely, was> 75% for all thresholds.

**Table 5:** Test parameters for MPR cut-offs in detecting unfavourable viral outcomes

MPR category	Number of individuals in category	Number of individuals with unfavourable outcomes	Sensitivity % (95% CI)	Specificity % (95% CI)	PPV % (95% CI)	NPV % (95% CI)	ROC^a^ area (95% CI)
< 0.80	97	21	65.6 (52.3–77.3)	12.2 (9.8–15.1)	6.8 (4.9–9.2)	78.4 (68.8–86.1)	0.39 (0.33–0.45)
< 0.90	137	24	60.7 (47.3–72.9)	18.2 (15.2–21.5)	6.8 (4.9–9.2)	82.5 (75.1–88.4)	0.39 (0.33–0.46)
< 0.95	197	31	49.2 (36.1–36.1)	26.7 (23.3–30.4)	6.2 (4.2–8.7)	84.3 (78.4–89.1)	0.38 (0.31–0.45)
< 1.00	346	44	27.9 (17.1–40.8)	48.6 (44.6–52.6)	5.1 (3.0–8.0)	87.3 (83.3–90.6)	0.38 (0.32–0.44)
< 1.05	510	52	14.8 (7.0–26.2)	73.8 (70.1–77.2)	5.2 (2.4–9.7)	89.8 (86.8–92.3)	0.44 (0.39–0.49)

^a^The average of sensitivity and specificity for the binary MPR cut-point being tested

*PPV* Positive predictive value, *NPV* Negative predictive value, *ROC* receiver operating characteristic

In the sensitivity analysis assessing transient rises as an unfavourable outcome, decreasing MPR was associated with a greater hazard ratio for non-transient rises (0.008) but not transient rises (See Appendix). Including transient rises had the impact of increasing the hazard ratios for unfavourable events above the cut point.

## Discussion

This is the largest study to date assessing the association between MPR and viral outcomes across multiple sites with single oral dose antiviral treatment for CHB using real world data [14, 19, 23]. Previous studies have evaluated viral outcomes with self-report or individual hospital cohorts [14, 16]. Approximately 80% of patients achieved suppression or were suppressed during the study period consistent with other studies [14, 24]. Lower MPR values were associated with unfavourable viral outcomes in people taking antiviral medication for CHB with an increased risk of viral breakthrough.

Values used for defining ‘poor adherence’ have previously been arbitrary and have ranged from 80 to 95% [7, 13, 19, 25–27], with a small study that examined entecavir and viral outcomes with a proposed cut-off of less than 90% [7]. This study illustrates that the hazard ratio for unfavourable outcomes increases with decreasing medication taking and that previous use of thresholds such as 0.90 are supported by evidence of a greater risk of events but are not a true cut-off points to define adherence based on viral outcomes. While self-reported patient adherence has recently also been shown to be associated viral outcomes, there was no clear dose response or increase in relative risk of viral breakthrough with increased doses reported to be missed [14], this finding is likely due to social desirability bias. Self-report, in a clinical context, has the advantage of a simple question to assess adherence however by using MPR or combining adherence measures in clinical practice it is possible to better assess the risk of unfavourable viral outcomes and advise patients how to mitigate that risk.

A small number of patients in this study remained on less effective therapies despite universal access to newer antiviral therapy as is also observed in national prescribing data for Australia [21]. Participants on older regimes did not have increased number of unfavourable outcomes despite the lower barrier to resistance observed with these agents. This may in part be due to small numbers, and possible selection bias as those remaining on older agents are likely to be those who had achieved and maintained virological suppression.

This study is limited by retrospective clinical data that are heterogeneous: the number of pharmacy visits, amount of drug dispensed at each visit and number of viral loads performed over the time-period all varied. No measure of patient adherence is without limitations [18]. Medication possession ratio calculated over any time-period masks variations in adherence: a period of stockpiling followed by a period of poor adherence, losing medication or conversely a period of poor adherence followed by regular medication taking. Calculation of MPR accounts for medication picked up from a pharmacy but does not equate to actual pill taking [18]. Oral antiviral therapy for the treatment of CHB was mainly dispensed via the hospital system during the time period so it is unlikely MPR was underestimated. It is possible that viral load test results could have been performed outside the hospital pathology service and not all viral events during the treatment period are captured.

The analysis was performed using real word data with different types of medication that may have different efficacies when evaluating viral suppression. Host immunological changes causing viral suppression, or the development of antiviral resistance while on medication could have influenced findings of undetectable viral load or viral breakthrough over and above medication adherence. This study was not powered to detect association with MPR and adverse outcomes including mortality as has been previously described in HIV therapy; this is in part due to the differing natural history of HBV and HIV, with very different time lines to attributable mortality off treatment.

Poor adherence is a challenge for health systems and all chronic diseases and there are few interventions that have been successful in improving adherence [17, 28]. Long term antiviral treatment for hepatitis B requires regular assessment of adherence by clinicians in a non-judgmental atmosphere [17]. Health systems need to enable ease of supply of medication, affordability of treatment and consistent practitioners that have a chance to build trust, knowledge and assist when they have challenges that may impact on adherence [13, 29].

Maximising outcomes for people living with CHB must include regular assessment of adherence and a focus on maximising good outcomes for people living with CHB. Adherence interventions have been rarely trialled in CHB and there is a need to explore further barriers faced by individuals and then design and implement appropriate interventions to assist the one in every five patients who have poor adherence [13].

## Conclusion

This study demonstrated that MPR is a useful measure to use to assess risk of poor viral outcomes. There is evidence for the 90% threshold commonly used to assess adherence, however there is a dose response relationship that must be considered in risk reduction. As we move towards increasing the number of people on treatment both in Australia and worldwide, evaluating hepatitis B viral suppression in multi centre or population-level data, and trialling systems improvement to enhance adherence will be increasingly important for achieving better health outcomes for people living with chronic hepatitis B.

## Data Availability

The datasets used and/or analysed during the current study are available from the corresponding author on reasonable request.

## Appendix

A further analysis was undertaken with the alternate classification of transient rises as an unfavourable outcome, as below:

**Table 6:** Viral outcome categories for HBV treatment used in sensitivity analysis

Favourable viral outcomes:	
Fully suppressed	HBV DNA undetectable for study period OR HBV DNA became undetectable with no further rise
Adequate viral suppression	Where treatment duration was < 2 years, HBV DNA decreased > 1 log10
Unfavourable viral outcomes:	
Not suppressed	Detectable HBV DNA after 2 years on treatment
Non-transient rise	< 1 log rise in HBV DNA which was not a transient rise
Viral breakthrough	≥1x log_10_ rise in HBV DNA level from nadir which was not a transient rise
Transient rise	a single instance of increased DNA level < 150 IU/ML with return to undetectable at next test

**Table 7:** Hazard ratio for unfavourable events for each MPR cut off threshold, and interpretation of risk reduction of unfavourable events, using alternate classifications presented in Table 6 in Appendix (sensitivity analysis)

MPR category	Number of individuals in category	Number of individuals with unfavourable outcomes	Hazard ratio for unfavourable events with MPR above threshold (95% CI) *p*-value	Interpretation of risk reduction of unfavourable events with MPR above threshold
< 0.80	97	11	0.08 (0.03–0.21) < 0.001	92%
< 0.90	137	12	0.11 (0.04, 0.30) < 0.001	89%
< 0.95	197	13	0.16 (0.05, 0.45) 0.001	84%
< 1.00	346	16	0.26 (0.07, 0.91) 0.034	74%
< 1.05	510	18	0.31 (0.04, 2.33) 0.254	No evidence of association

## Abbreviations

CHB: Chronic hepatitis B
CI: Confidence interval
HBV: Hepatitis B virus
HBV: DNA Hepatitis B virus DNA or viral load
HIV: Human immunodeficiency virus
IQR: Inter-quartile range
MPR: Medication possession ratio
NPV: Negative predictive value
PPV: Positive predictive value
ROC: Receiver operating characteristic
SD: Standard deviation
